# Effects of personality traits on bus drivers’ prosocial and aggressive behaviours: The moderated mediating role of risk perception and gender

**DOI:** 10.1371/journal.pone.0281473

**Published:** 2023-02-07

**Authors:** Sihui Wang, Youran Zhang, Long Sun

**Affiliations:** 1 School of Psychology, Liaoning Normal University, Dalian, China; 2 School of Psychological and Cognitive Sciences, Peking University, Beijing, China; Al Mansour University College-Baghdad-Iraq, IRAQ

## Abstract

**Purpose:**

The present study aimed to examine the effects of personality traits on bus drivers’ self-reported prosocial and aggressive driving behaviours as well as the mediating role of risk perception and the moderating role of gender in this relationship.

**Methods:**

Three hundred and ten bus drivers who were 24–57 years old agreed to participate in this study. The measures utilized included personality scales, a risk perception scale and a prosocial and aggressive driving behaviour scale.

**Results:**

A moderated mediation model was established. The effects of normlessness/anger on prosocial driving behaviour are mediated by risk perception. Risk perception has a stronger promoting effect on the prosocial driving behaviour of male drivers (*b* = 0.358, *p* < 0.01) than it does on that of female drivers (*b* = 0.072, *p* > 0.05). The effects of normlessness/anger on the aggressive driving behaviour of both male and female drivers are also mediated by risk perception. Moreover, gender plays a moderating role in the influences of personality and risk perception on aggressive driving behaviour. Drivers with higher risk perception and less anger exhibit less aggressive driving behaviour, and this effect is clearer among male drivers than it is among female drivers.

**Conclusion:**

The present study revealed the relationship between personality traits and the prosocial and aggressive driving behaviours of bus drivers through a moderated mediation model. These findings highlight the importance of taking risk perception and gender into consideration when examining the effects of personality on bus drivers’ driving behaviours.

## 1. Introduction

A newly released report shows that the number of buses in China is about 2.34 million in 2020 and there were 228 traffic accidents involving buses, causing 33 deaths and 214 injuries, in the six months from December 2020 to May 2021 [1]. Most traffic accidents are associated with aggressive driving behaviours, including drivers explicitly acting aggressively and deliberately violating traffic regulations without expressing aggressive motives [2]. Given that buses are the most commonly used means of public transportation in many countries, studies concerning aggressive driving behaviour and its influencing factors among bus drivers have attracted much interest [3–8]. However, most of these studies have focused on aggressive driving behaviours or crash risk, and little attention has been given to bus drivers’ prosocial driving behaviours.

Prosocial driving behaviour is a safe driving behaviour that can protect the safety and health of other road users and promote effective cooperation with others in a driving environment [8]. Previous studies have found that prosocial or safe driving behaviour is a significant predictor of private car drivers’ crash involvement [9,10]. For example, one study found that drivers’ prosocial driving behaviours were significantly associated with their penalty points and fines received in the last year [11]. To date, only one study has examined the safe and risky driving behaviours of professional drivers (i.e., truck and bus drivers) and non-professional drivers [12]. This study found that safe behaviour can significantly predict professional drivers’ crash involvement. However, non-professional drivers’ crash involvement was significantly associated with ordinary and aggressive violations but not with safe driving behaviours [12]. Other studies compared the differences in driving behaviour between professional drivers and non-professional drivers, but the instruments used in these studies usually targeted risky driving behaviours, such as speeding [13], aggression [14] and lapses, errors, violations [15]. These findings suggest that encouraging safe driving behaviours might provide an alternative way to reduce bus drivers’ traffic accidents.

### 1.1 Personality traits and driving behaviours

Although many human factors account for risky or aggressive driving behaviour, most of the studies in the related body of literature have focused on personality traits, such as altruism, sensation seeking and anger [4,5,16–19]. In contrast to other personality traits, such as locus of control [17], these personality traits have been proven to be effective and stable predictors of aggressive driving behaviour [11]. Generally, the higher drivers score on sensation-seeking, anger and normlessness scales, the more aggressive driving behaviours they exhibit and the more traffic violations they have had in the past [4,17]. For example, one study found that drivers with high anger scores are prone to emotional instability, anger and frustration, and in turn, they exhibit aggressive driving behaviours [19]. In addition, personality traits can directly and significantly predict aberrant driving behaviours among bus drivers [4]. Professional drivers’ altruism was found to be negatively correlated with aberrant driving behaviours such as aggressive and ordinary violations [5]. These findings have played an important role in reducing traffic casualties. However, to the best of our knowledge, no studies have examined the effects of personality traits (normlessness, sensation-seeking, altruism, anger) and their interactions with other factors, such as risk perception and gender, on bus drivers’ aggressive driving behaviours.

### 1.2 The mediating role of risk perception

Risk perception refers to the perceived probability of road traffic accidents and the perceived potential severity of their consequences [20]. Previous studies have found that drivers who score low on risk perception scales are associated with increases in the number of road accidents and risky driving behaviours [20–22]. Moreover, three studies have examined the mediating role of risk perception in the relationship between personality and risky driving behaviours [22,23]. For example, Machin and Sankey found that risk perception mediates the relationships between young drivers’ sensation-seeking, altruism and speeding behaviours [22]. Although these studies have revealed relationships between private car drivers’ personalities, risk perceptions and driving behaviours, the effects of personality and risk perception on the aggressive or prosocial driving behaviours of bus drivers have not been extensively explored. Additionally, whether risk perception mediates the relationship between the personalities and driving behaviours of bus drivers is still unknown.

**Fig 1:**
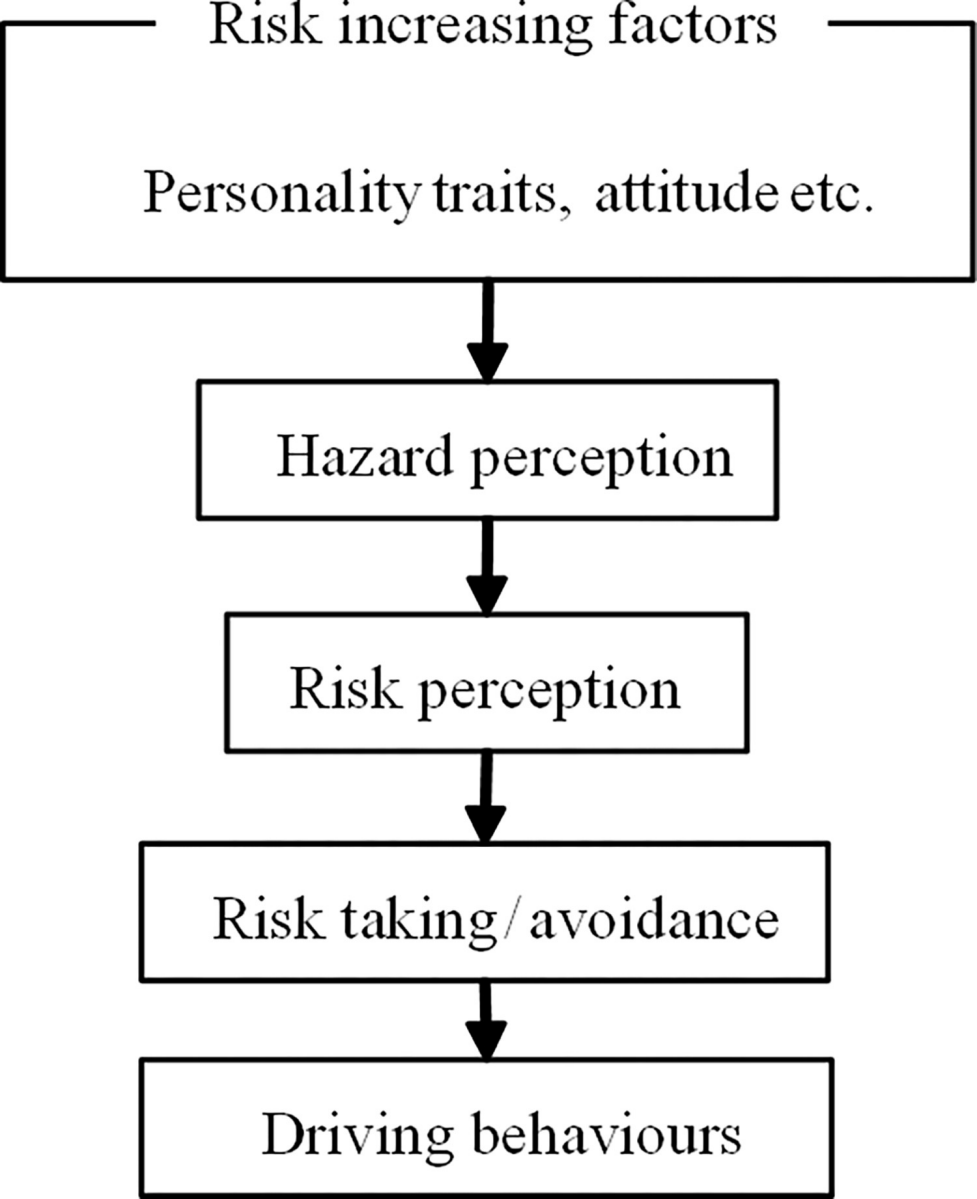
Driving behaviour model based on risk perception [24].

This study applied the driving behaviour model of risk perception (Fig 1) to examine the mediating role that risk perception plays in the relationship between personality traits and driving behaviours [24]. As illustrated in Fig 1, this model demonstrated that a driver determines which driving behaviour to adopt after he or she evaluates the current road risk situation. Personality traits were shown to affect driving behaviour indirectly, and the effects of personality traits on driving behaviours were mediated by risk perceptions. The mediating role of risk perception has been supported by the findings of several studies [18,22,25,26]. Hence, risk perception is used as a mediating factor in the meditation model of the present study.

On the other hand, studies concerning this theoretical framework have mainly focused on private car drivers’ risk-taking behaviours [27]. Given that prosocial and aggressive driving behaviours are more universal than risk-taking behaviours, it is of great importance to concurrently examine bus drivers’ safe and unsafe driving behaviours and deeply investigate their associations with personality and risk perceptions.

### 1.3 The moderating role of gender

Compared to other sociodemographic variables such as age and years of driving experience, gender is shown to be a strong predictor of aggressive driving behaviour in published studies [3,26,28]. Specifically, male drivers are more likely than female drivers to engage in risky driving behaviour [18] or adopt unsafe driving styles [10,29].

More importantly, some studies have found that drivers’ risk perceptions are related to gender, and they have demonstrated that a private car driver’s gender plays a moderating role in the effect of risk perception on risky driving behaviour [30] or in the relationship between PTSD symptoms and aberrant driving behaviour [31]. For instance, female drivers are found to report higher levels of risk associated with risky driving behaviour than male drivers [30]. A few studies have also shown that the interaction between personality and gender affects driving performance [16,26]. Song et al. [26] found that the effects of driving experience on risky driving behaviours are mediated by sensation seeking and moderated by gender. However, studies concerning the effects of these factors on bus drivers’ traffic safety are still rare. Thus, the present study further examines whether the relationships between bus drivers’ personality traits, risk perceptions and driving behaviours are affected by gender.

### 1.4 Aim of the present study

**Fig 2:**
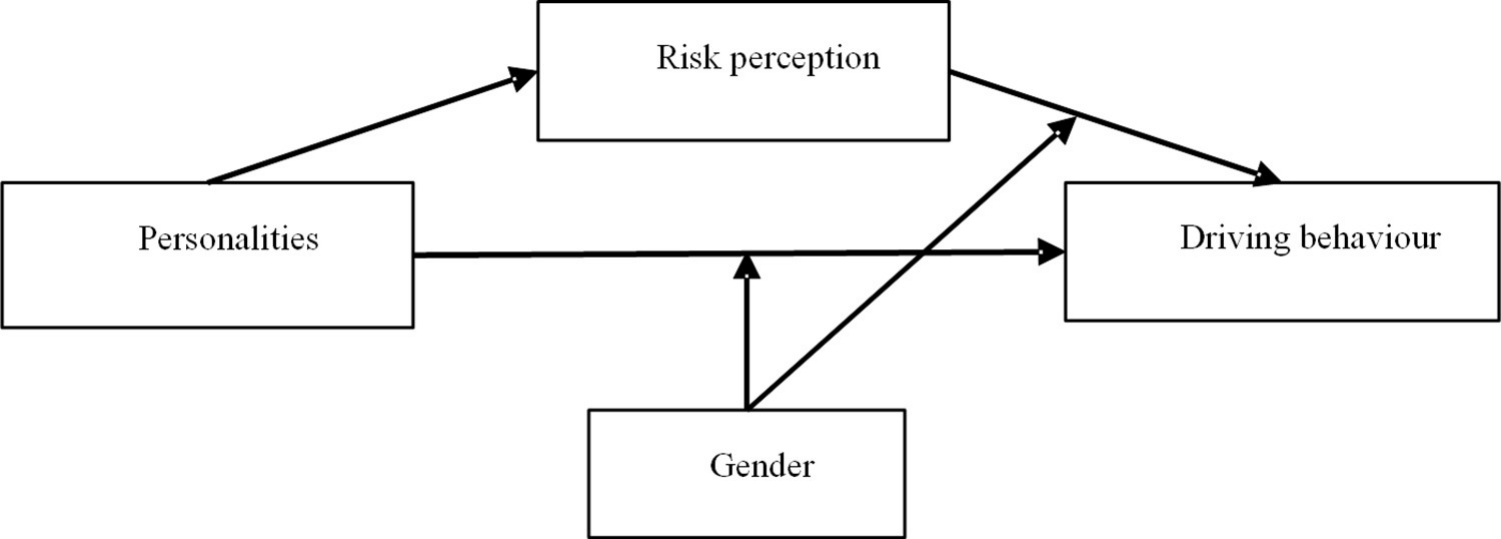
The proposed theoretical model.

The present study aimed to examine the effects of personality traits on bus drivers’ self-reported prosocial and aggressive driving behaviours as well as the mediating role of risk perception and the moderating role of gender in these relationships. The previously proposed relationships between personality traits, risk perception, driving behaviours and gender are shown in Fig 2. As illustrated in Fig 2, the predictor variables (personality traits), the mediating variable (risk perception), moderating variable (gender) and the result variable (prosocial and aggressive driving behaviours) were presented.

The present study hypothesizes that:

H1: Risk perception mediates the relationship between personality traits and driving behaviours;
H2: Gender moderates the relationship between personality traits and driving behaviours;
H3: Gender also moderates the relationship between risk perception and driving behaviours.

## 2. Methods

### 2.1 Participants and procedure

This study was approved by the Logistics Department for Civilian Ethics Committee of Liaoning Normal University. The participants included in the sample were recruited randomly from three bus companies located in Shanghai (southeastern China), Dalian (northeastern China) and Kunming (southwestern China), which are geographically represent Chinese cities. A total of 330 participating drivers were informed about the purpose of the study, and pencil-and-paper procedures were administered to 310 drivers who were willing to take part in this survey. The written informed consent was obtained from all participants for inclusion in the study. The participants were asked to answer several 30-minute questionnaires between October 1 and October 15 of 2019 anonymously. Each participant received a gift (10-yuan RMB) after completing the survey.

The final data set comprised 294 valid responses, yielding an effective response rate of 94.8%. Sixteen participants were excluded from the sample due to some missing responses. The sample included 174 male drivers (59.2%) and 120 female drivers (40.8%). The participants’ ages ranged from 24 to 57 years with a mean age of 37.10 (*SD* = 5.95). The length of the participants’ driving experience ranged from 2 to 30 years (*SD* = 6.64). Additionally, 31.0% of the participants had a middle school education, 55.1% had a high school education, and 13.9% had a college education. Table 1 shows the detailed demographic information of the participants.

**Table 1:** Demographic information.

Variables	*n*		Percent (100%)	
	Male	Female	Male	Female
24~34 years old	62	44	58.5	41.5
35~44 years old	92	65	58.6	41.4
Over 45 years old	20	11	64.5	35.5
Driving experience under 5 years	23	17	57.5	42.5
5–10 years’ driving experience	39	59	39.8	60.2
11~15 years’ driving experience	35	22	61.4	38.6
16~20 years’ driving experience	52	10	83.9	16.1
Driving experience more than 21 years	25	12	67.6	32.4

### 2.2 Measures

Measurements included Prosocial and aggressive driving behaviour Inventory (PADI), personality traits, risk perception and demographic information were administrated to each participant.

#### 2.2.1 PADI

The Chinese version of the PADI has 29 items and includes two dimensions [11], which was initially developed by Harris et al. [9] Aggressive behaviour (17 items) refers to drivers intentionally violating traffic rules or using unsafe manoeuvres to express an unpleasant mood. Prosocial behaviour (12 items) refers to a driver’s initiative to give way to other vehicles and cooperate well with other road users. Participants were asked to rate each item on a 6-point Likert scale ranging from “never (1)” to “always (6)”. In this study, the internal reliability of the aggressive driving behaviour is 0.68, and 0.90 for prosocial driving behaviours.

#### 2.2.2 Personality scales

The personality scales consist of 4 traits and have been widely used in relevant studies concerning road safety in China [19]. The 4 traits are anger (10 items), sensation-seeking (10 items), altruism (10 items) and normlessness (4 items). The items of the first three traits came from the International Personality Item Pool (IPIP, http://ipip.ori.org) [32] and the items of normlessness came from Kohn and Schooler [33]. Participants answered the items on a 5-point Likert scale ranging from “strongly disagree (1)” to “strongly agree (5)”.

Anger refers to the tendency to perceive a wide range of situations as frustrating or irritating. Sensation-seeking refers to the desire to seek stimulation and novel experiences. Altruism refers to the tendency to be cooperative, kind-hearted, and concerned about others. Normlessness refers to adopting unacceptable behaviours to achieve certain goals. In this study, the internal reliability of anger is 0.69, 0.71 for sensation-seeking, 0.65 for altruism and 0.69 for normlessness.

#### 2.2.3 Risk perception

The scale contained 6 items that were derived from an existing survey developed by Iversen and Rundmo [20]. First, participants were asked to express how worried and concerned they were about being hurt in a traffic accident (3 items), ranging from “not worried at all (1)” to “very worried (7)”. Second, they were asked to rate their probability of being involved in a traffic accident in the future (3 items), ranging from “not probable at all (1)” to “very probable (7)”. The total score was obtained by averaging the 6 items. In the present study, the internal reliability of risk perception is 0.67.

#### 2.2.4 Demographic questionnaire

Participants were also asked to provide information about their gender, age, years of driving experience and years of education.

### 2.3 Data analysis

The data were analysed using SPSS 18. First, common method bias among the measures was examined using Harman’s single-factor method [34]. Second, the differences in these behaviours by gender and other demographic factors were further examined with univariate ANOVAs and Pearson correlations. Third, Pearson correlations were calculated for the relationships between prosocial and aggressive driving behaviours, personality and risk perception. Finally, we conducted a moderated mediation analysis as suggested by Preacher et al. [35] using the PROCESS syntax [36]. Notably, only those demographic variables whose effects are significant in the MANCOVA will be examined as moderating factors.

### 2.4 Common method bias

This study assessed common method bias using Harman’s single-factor method [31]. Specifically, the first factor accounted for 14.29% of the variance, and factors whose eigenvalues exceeded 1.0 accounted for 63.81% of the variance. Thus, common method bias does not appear to be a serious problem for this study.

## 3. Results

### 3.1 Effects of demographic variables on driving behaviours

Univariate ANOVAs were first conducted to compare the differences in the means of male and female bus drivers, and the results are shown in Table 2.

**Table 2:** Gender differences in prosocial and aggressive driving behaviours (*M±S*D).

Driving behaviours	Male	Female	*F* (1,293)	Partial *η2 p*
Prosocial behaviour	5.53 ± 0.59	5.70 ± 0.27	13.28**	0.029
Aggressive behaviour	2.68 ± 0.82	2.49 ± 0.59	5.69*	0.016

Note: *p< 0.05, ** *p*< 0.01.

Table 2 shows that female drivers’ scores on prosocial driving behaviour are higher than those of male drivers, while their scores on aggressive driving behaviour are lower than those of male drivers. Pearson correlation analysis shows that the associations between age, driving experience, education and prosocial and aggressive driving behaviours were not significant.

### 3.2 Correlation analysis

The correlations between personality traits, risk perception and driving behaviours are shown in Table 3.

**Table 3:** Correlations between risk perception, personality traits and driving behaviours.

Variables	1	2	3	4	5	6	7	8	9	10	11
1 Gender	-										
2 Age	-0.00	-									
3 Experience	-0.24**	0.75**	-								
4 education	0.04	-0.03	-0.03	-							
5 Normlessness	0.00	-0.02	-0.02	0.02	-						
6 Sensation-seeking	-0.09	0.01	0.03	-0.03	0.18**	-					
7 Altruism	0.10	-0.08	-0.04	-0.00	-0.29**	-0.26**	-				
8 Anger	-0.01	0.10	0.04	0.08	0.21**	0.11	-0.31**	-			
9 Risk perception	0.02	0.04	0.06	0.00	-0.19**	-0.08	0.12*	-0.17**	-		
10 Prosocial behaviour	0.17**	0.00	0.02	0.08	-0.26**	-0.19**	0.28**	-0.22**	0.30**	-	
11 Aggressive behaviour	-0.13*	-0.03	-0.02	-0.08	0.40**	0.22**	-0.30**	0.31**	-0.58**	-0.45**	-

Note: *p< 0.05, ** *p*< 0.01.

Table 3 shows that prosocial driving behaviours are negatively and significantly correlated with normlessness, sensation seeking and anger and positively correlated with altruism and risk perception, while aggressive driving behaviour is positively and significantly correlated with anger, sensation seeking and normlessness and negatively correlated with altruism and risk perception.

### 3.3 Moderated mediation model

**Fig 3:**
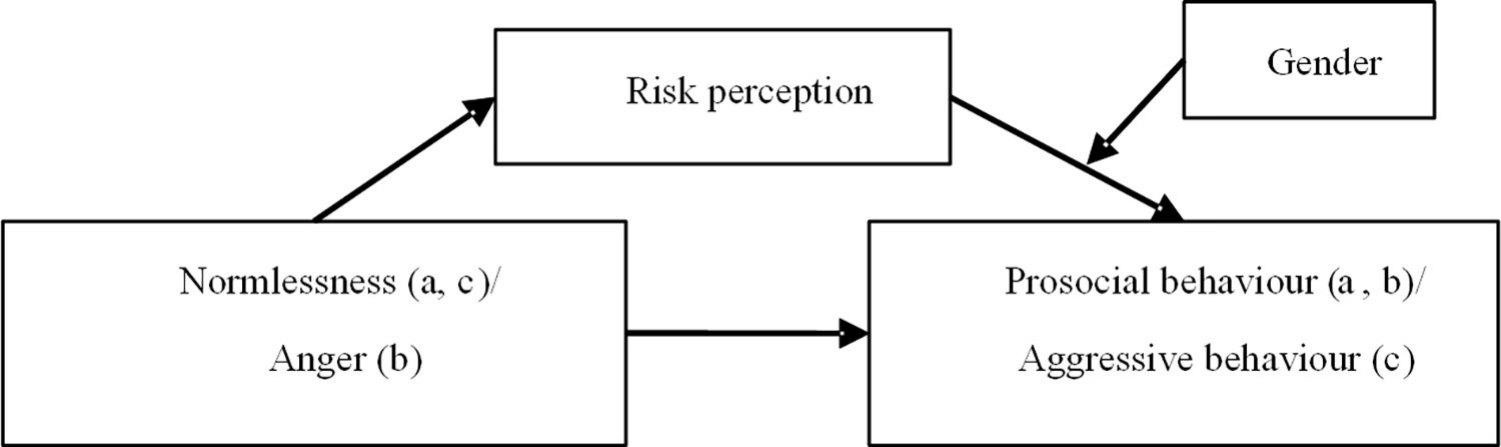
Model a, b, c. Note: Model a shows the effect of normlessness on prosocial driving behaviour, Model b shows the effect of anger on prosocial driving behaviour, and Model c shows the effect of normlessness on aggressive driving behaviour.

**Fig 4:**
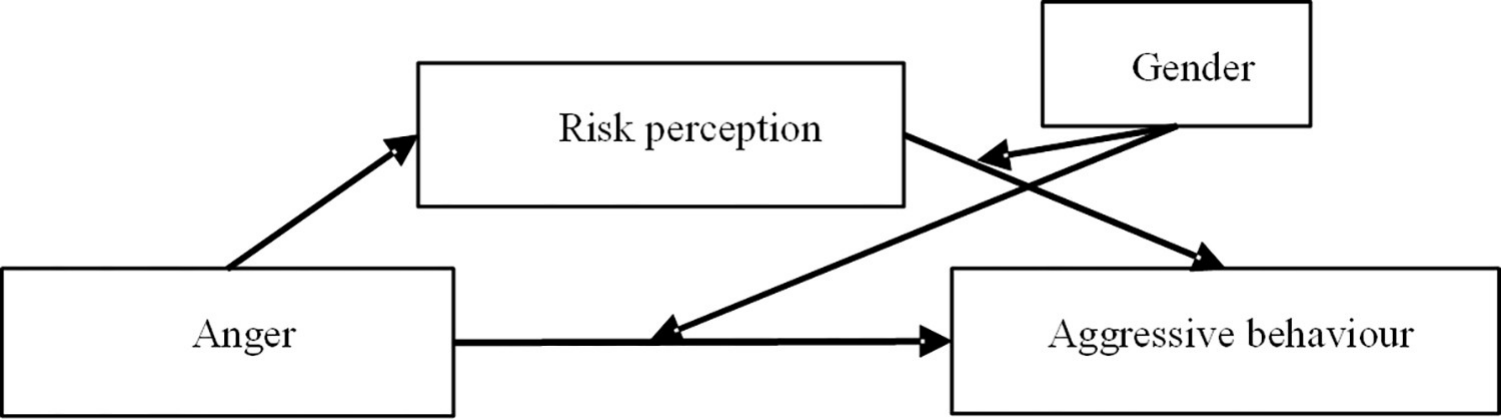
Model d: The effect of anger on aggressive driving behaviour.

The models developed in this study are shown in Figs 3 and 4, and the indirect effects of personality traits on prosocial and aggressive driving behaviours (mediated by risk perception and moderated by gender) were proven in the present study.

This study used Models 14 and 15 of the SPSS macro compiled by Hayes et al. [36], and the results showed a significant mediating effect of risk perception on the relationship between the examined personality traits (normlessness and anger) and driving behaviours. Hypothesis H1 was supported. Moderated mediation results of personality traits on prosocial driving behaviour are shown in Table 4.

**Table 4:** Moderated mediation results of personalities on prosocial driving behaviour.

Variables	Risk perception			Prosocial driving behaviour		
	Coeff.	SE	*t*	Coeff.	SE	*t*
Constant	5.239	0.097	54.250**	2.778	0.673	4.129**
Normlessness	-0.128	0.039	-3.253**	-0.109	0.030	-3.596**
Risk perception				0.573	0.132	4.330**
Gender				1.437	0.442	3.247**
Risk perception × Gender				-0.257	0.089	-2.895**
*R^2^*	0.035			0.185		
*F*	10.579			16.342		
Constant	5.535	0.206	26.925**	2.775	0.680	4.083**
Anger	-0.177	0.061	-2.909**	-0.151	0.047	-3.246**
Risk perception				0.626	0.132	4.753**
Gender				1.599	0.443	3.615**
Risk perception × Gender				-0.290	0.089	-3.269**
*R^2^*	0.028			0.178		
*F*	8.464			15.639		

Note

*p< 0.05

** *p*< 0.01.

Moderated mediation results of personality traits on aggressive driving behaviour are shown in Table 5.

**Table 5:** Moderated mediation results of personalities on aggressive driving behaviour.

Variables	Risk perception			Aggressive driving behaviour		
	Coeff.	SE	*t*	Coeff.	SE	*t*
Constant	5.239	0.097	54.250**	7.446	0.832	8.954**
Normlessness	-0.128	0.039	-3.253**	0.241	0.037	6.436**
Risk perception				-1.041	0.164	-6.359**
Gender				-1.610	0.547	-2.944**
Risk perception × Gender				0.290	0.110	2.643**
*R^2^*	0.035			0.445		
*F*	10.579			57.993		
Constant	5.535	0.206	26.925**	6.050	1.112	5.443**
Anger	-0.177	0.061	-2.909**	0.674	0.183	3.688**
Risk perception				-1.101	0.168	-6.557**
Gender				-0.865	0.738	-1.173**
Anger × Gender				-0.266	0.117	-2.266**
Risk perception × Gender				0.319	0.113	2.812**
*R^2^*	0.028			0.422		
*F*	8.464			42.119		

Note

*p< 0.05

** *p*< 0.01.

In addition, gender was shown to moderate the relationships between risk perception and the two driving behaviours and the relationship between anger and aggressive driving behaviours. Hypotheses H2 and H3 were also supported.

As suggested by Preacher et al. [35] and Sarbescu et al. [37] a bootstrap confidence interval (95% CI) that excludes 0 indicates a significant effect. The data were decentralization prior to analysis. The results are shown in Table 6.

**Table 6:** Results of the indirect effect analysis.

	Sex	Effect	BootSE	BootLLCI	BootULCI
Model a	Male	-0.041	0.021	-0.088	-0.006
	Female	-0.008	0.005	-0.017	0.002
Model b	Male	-0.060	0.027	-0.115	-0.012
	Female	-0.008	0.006	-0.021	0.004
Model c	Male	0.096	0.041	0.028	0.188
	Female	0.059	0.025	0.019	0.117
Model d	Male	0.139	0.060	0.033	0.269
	Female	0.082	0.037	0.020	0.167

Note

*p< 0.05

** *p*< 0.01.

Table 6 shows that for both the male and the female drivers, Model c (*b* = 0.096, 95% CI [0.028, 0.188]; *b* = 0.059 and 95% CI [0.019, 0.117]) and Model d (*b* = 0.139, 95% CI [0.033, 0.269]; *b* = 0.082, 95% CI [0.020, 0.167]) were supported. In addition, for the male drivers, Model a (*b* = -0.041 and 95% CI [-0.088, -0.006]) and Model b (*b* = -0.060, 95% CI [-0.115, -0.012]) were feasible.

**Fig 5:**
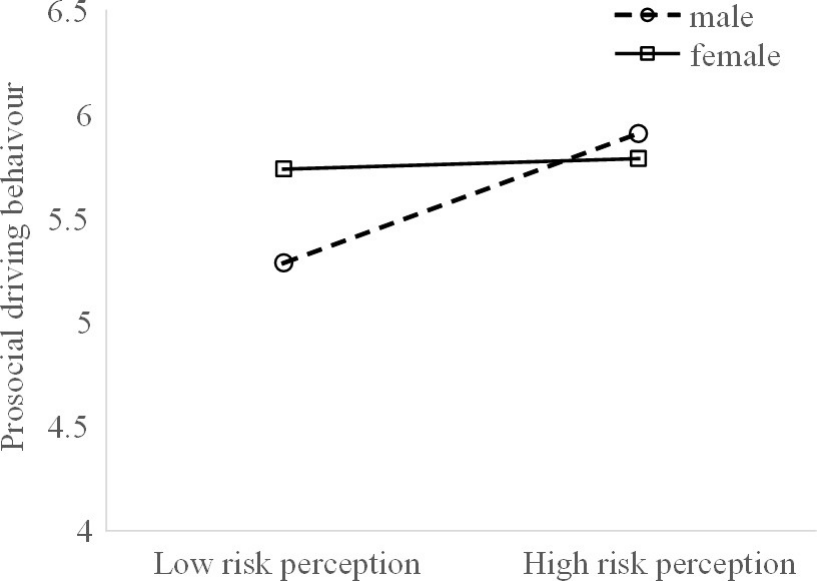
Simple slope analysis of prosocial behaviours-gender × risk perception.

A further simple slope analysis showed that (Fig 5) the link between risk perception and prosocial driving behaviour was significant only for the male drivers (*b* = 0.358, *p* < 0.01). That is, a higher level of risk perception was associated with increased prosocial behaviour for the examined male bus drivers. However, this effect was not significant for the female drivers (*b* = 0.072, *p* > 0.05).

**Fig 6:**
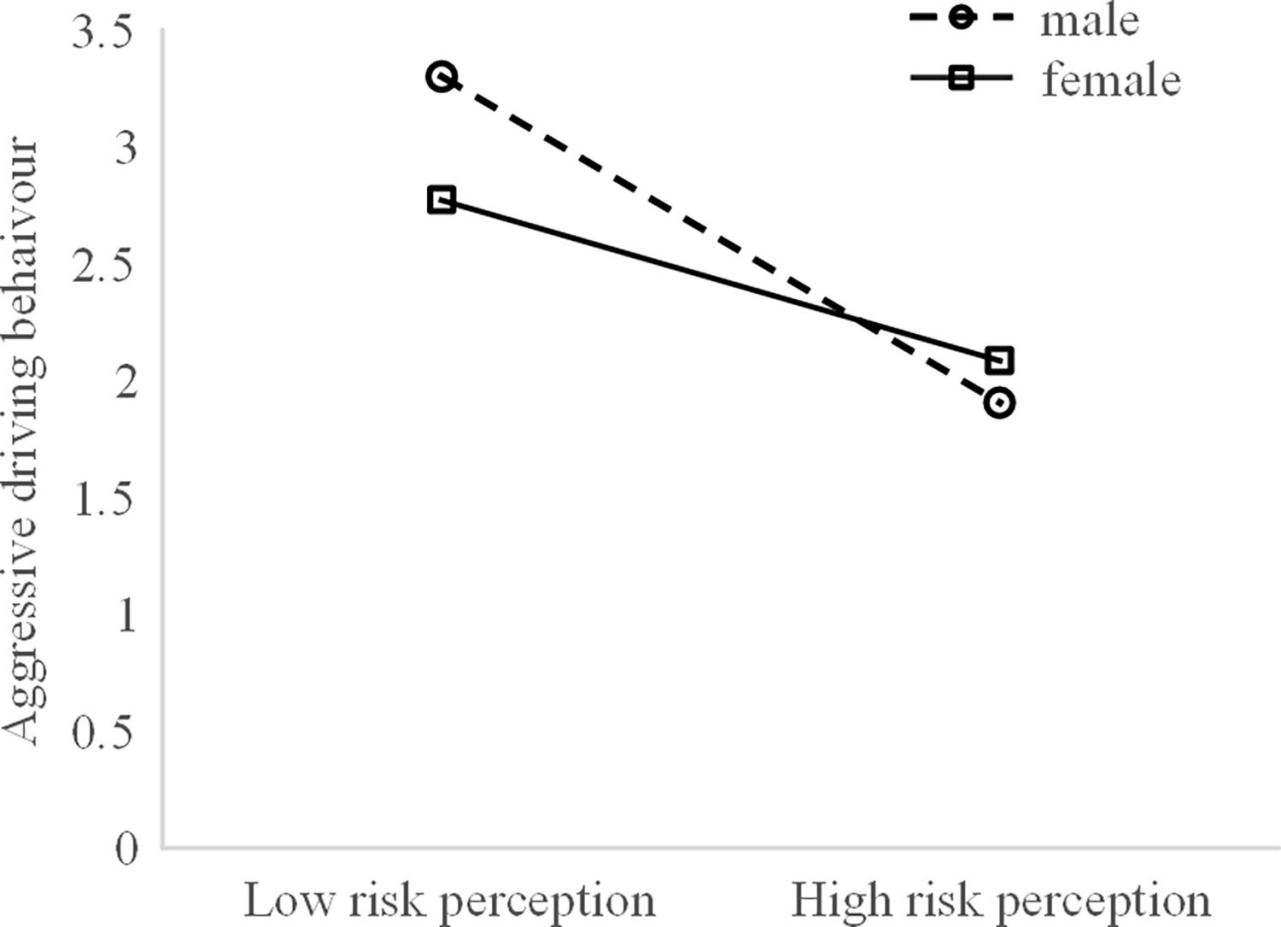
Simple slope analysis of aggressive behaviours-gender × risk perception.

As shown in Fig 6, the link between risk perception and aggressive driving behaviour was significant for both the male (*b* = -0.842, *p* < 0.01) and the female (*b* = -0.488, *p* < 0.01) drivers. In addition, the slope was steeper for the male drivers.

**Fig 7:**
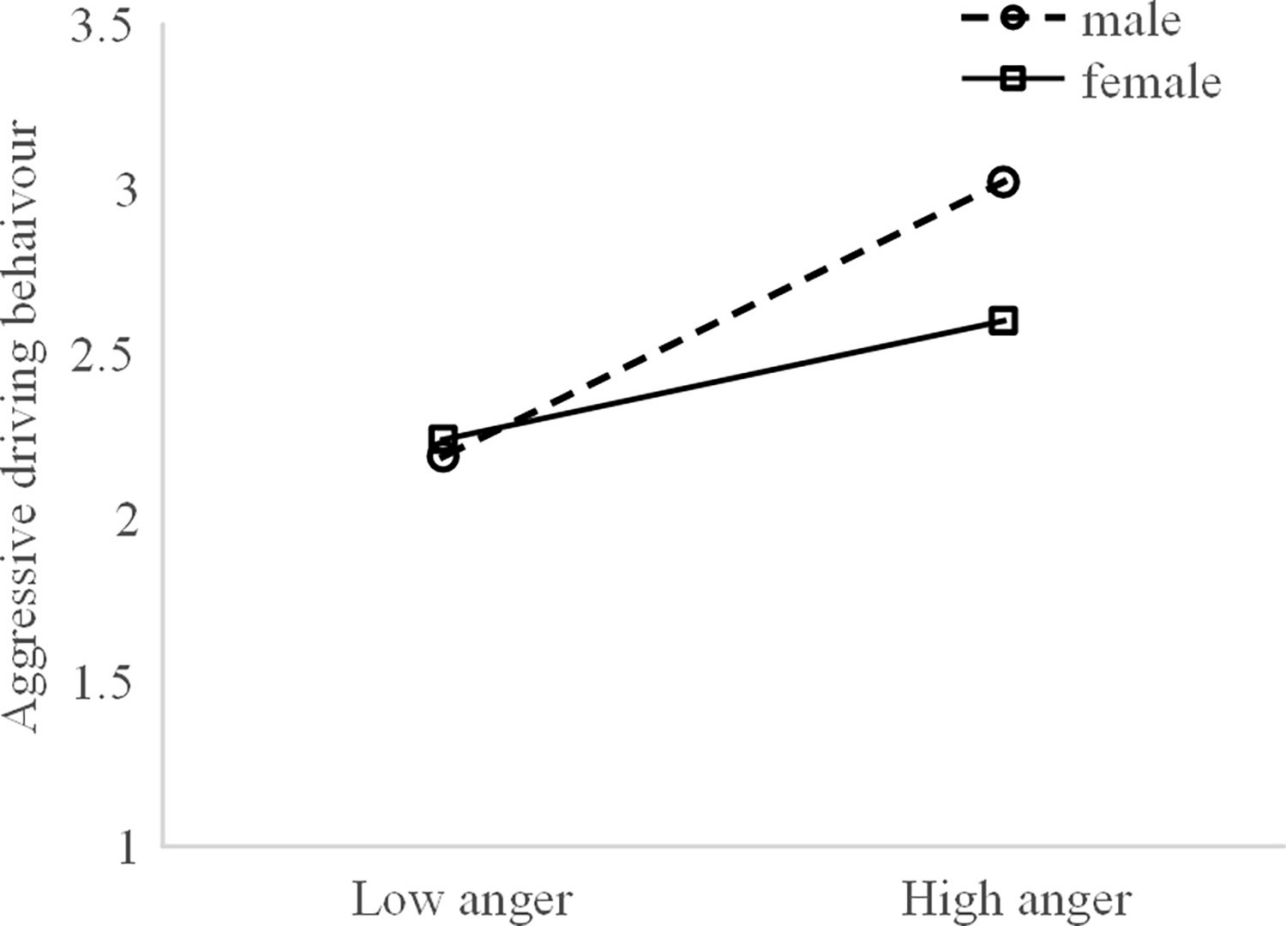
Simple slope analysis of aggressive behaviours-anger × gender.

As shown in Fig 7, the link between anger and aggressive driving behaviour was pronounced for both the male (*b* = 0.555, *p* < 0.01) and the female (*b* = 0.218, *p* < 0.05) drivers. This pattern was more pronounced among the male drivers.

## 4 Discussion

The present study found that personality trait, risk perception, and gender, as well as the interactions among these factors, influence bus drivers’ driving behaviours, providing evidence for the driving behaviour model of risk perception [24]. We find that lower scores on altruism, risk perception and higher scores on normlessness, sensation seeking and anger are associated with decreased prosocial driving behaviours and increased aggressive driving behaviours. These replicated the findings of some previous studies with private car drivers [20–22]. Importantly, risk perception mediates the relationships between personality traits and driving behaviour, and gender moderates the relationships between risk perception, personality traits and driving behaviour. The findings improve our understanding of both prosocial and aggressive driving behaviours among bus drivers and highlight the importance to integrate personality traits and risk perception together when considering driving safety.

### 4.1 The mediating role of risk perception

This study verifies that risk perception is a potential mediator of the influence of personality traits on prosocial and aggressive driving behaviours. We analyse the reasons that normlessness and anger improve aggressive driving behaviours through our model. It is possible that highly normless and angry bus drivers’ perceptions of road risk may be relatively careless and undetailed. As a result, such drivers may exhibit low risk perception, aggressive driving behaviours and few prosocial driving behaviours [18]. However, the present study shows that the adverse effects of normlessness and anger on driving behaviours can be alleviated through risk perception.

Furthermore, this study finds that risk perception plays a mediating role in the relationship between normlessness/anger and prosocial driving behaviours. However, these results are only found among male bus drivers. It is found that increased levels of normlessness and anger are associated with fewer prosocial driving behaviours among male drivers. Our results also show that a higher score on a risk perception scale is associated with more prosocial driving behaviours. A possible reason for this result is cultural differences [8]. Positive safety cultures may reduce aggressive driving violations in China. Another explanation is that this study aims to explore the relationship between personality traits and driving behaviours among bus drivers rather than private car drivers. It is acknowledged that bus drivers must undergo much safety training before they begin to drive professionally, making their safety awareness and risk perception better than those of private car drivers. Previous studies have demonstrated that risk perception mediates the relationships between personality and speeding behaviours [22] and personality and the frequency of mobile phone use [23].

### 4.2 The moderating role of gender

The effects of personality traits and their interactions with gender on driving behaviours are further discussed in the context of the moderated mediation model. First, gender is shown to moderate the relationship between risk perception and prosocial driving behaviours. Risk perception affects male drivers more strongly than female drivers in terms of prosocial driving behaviour. This may be due to the different risk perception abilities of male and female drivers. It has been found that such differences in risk perception lead to an increased level of risky driving behaviours among young and male drivers [30]. Another possible reason for this phenomenon is that female drivers are more likely to engage in prosocial driving behaviours, as is found in the present study as well as some previous studies [3,11].

Gender also moderates the relationship between risk perception and aggressive driving behaviour. Regardless of gender, a lower level of risk perception increases aggressive driving behaviours. However, this effect is clearer among male drivers than it is among female drivers. These results replicate the findings of previous studies showing that risky driving behaviours are associated with low scores on risk-perception scales [20–22].

Notably, this study finds that the effect of anger on aggressive driving behaviour is also moderated by gender. Although more anger leads to more aggressive driving behaviour, this effect is more profound for male drivers than it is for female drivers. Previous studies have found that male drivers are more aggressive than female drivers and more prone to anger and irritability, a lack of self-control and risky driving behaviours [3,10]. These results suggest that psychological interventions targeting the anger trait can be developed to enhance the driving safety of bus drivers.

### 4.3 Practical implications and limitations

This study explores the effects of the relationships between personality traits, risk perception and gender on the prosocial and aggressive driving behaviours of bus drivers. The moderated mediating model established in this study is the first to include both aggressive and prosocial driving behaviours, providing a new theoretical framework for explaining bus drivers’ safe and unsafe driving behaviours. These results have practical implications. First, understanding the influencing factors associated with bus drivers’ prosocial and aggressive driving behaviours may help develop interventions in order to increase traffic safety. Notably, interventions and efforts targeted at reducing aggressive driving behaviours or encouraging prosocial driving behaviours should take the effect of gender into consideration. Second, this study finds that a high level of risk perception can not only directly improve safe driving behaviours but also mitigate the negative impacts of personality traits such as normlessness and anger on driving safety. Given that personality is stable, risk perception-related training or interventions could be used as an alternative way to ensure the driving safety of bus drivers.

This study has some limitations. One limitation is that the participants of the present study do not constitute a representative sample of Chinese bus drivers, which limits the generalizability of this study to all Chinese bus drivers. Another limitation is that data of self-reported aggressive driving behaviours were collected. The relationships between aggressive driving behaviours and personality, risk perception might be underestimated due to social desirability bias. It should be noted that self-reported aggressive driving behaviours and on-road aggressive behaviours are often not the same [6]. When bus drivers respond to questions regarding their own aggressive driving behaviours, they often tend to undervalue the aggressive nature of their driving, especially when their livelihood might depend on their driving performance. Future studies should further investigate the relationships between personality traits, risk perception and on-road aggressive and prosocial driving behaviours, thus validating the present findings. A third limitation is that except for the variables mentioned in this study, there are also some important environmental factors that affect risky driving, such as work shifts [38], previous penalties [39] and the amount of work traffic in the service area [40]. Future research should further explore the influences of bus driver’s own characteristics and environmental factors on their driving behaviours.

### 5. Conclusions

The present study revealed the relationship between personality traits and the prosocial and aggressive driving behaviours of bus drivers through a moderated mediation model. This study not only emphasizes the importance of including the mediating role of risk perception in studies concerning personality traits and bus drivers’ driving behaviour but also highlights the moderating role of gender in such models. The findings emphasize the need to consider the combined effects of personality traits, risk perception and gender when interventions and education programmes are developed for bus drivers.
